# Silicon enhances the drought resistance of peach seedlings by regulating hormone, amino acid, and sugar metabolism

**DOI:** 10.1186/s12870-022-03785-5

**Published:** 2022-09-01

**Authors:** Huaifeng Gao, Wenying Yu, Xiaoqing Yang, Jiahui Liang, Xiwu Sun, Maoxiang Sun, Yuansong Xiao, Futian Peng

**Affiliations:** State Key Laboratory of Crop Biology, College of Horticulture Science and Engineering, Shandong Agricultural University, Tai-An, 271018 China

**Keywords:** Silicon, Drought, Peach, Hormone, Amino acid, Sugar

## Abstract

**Background:**

Drought is one of the main concerns worldwide and restricts the development of agriculture. Silicon improves the drought resistance of plants, but the underlying mechanism remains unclear.

**Results:**

We sequenced the transcriptomes of both control and silicon-treated peach seedlings under drought stress to identify genes or gene networks that could be managed to increase the drought tolerance of peach seedlings. Peach (*Prunus persica*) seedlings were used to analyse the effects of silicon on plant growth and physiological indexes related to drought resistance under drought stress. The results showed that silicon addition improved the water use efficiency, antioxidant capacity, and net photosynthetic rate, inhibition of stomatal closure, promoted the development of roots, and further regulated the synthesis of hormones, amino acids and sugars in peach seedlings. A comparative transcriptome analysis identified a total of 2275 genes that respond to silicon under drought stress. These genes were mainly involved in ion transport, hormone and signal transduction, biosynthetic and metabolic processes, stress and defence responses and other processes. We analysed the effects of silicon on the modulation of stress-related hormonal crosstalk and amino acid and sugar metabolism. The results showed that silicon promotes zeatin, gibberellin, and auxin biosynthesis, inhibits the synthesis of abscisic acid, then promote lateral root development and inhibit stomatal closure, and regulates the signal transduction of auxin, cytokinin, gibberellin and salicylic acid. Silicon also regulates the metabolism of various amino acids and promotes the accumulation of sucrose and glucose to improve drought resistance of peach seedlings.

**Conclusions:**

Silicon enhanced the drought resistance of peach seedlings by regulating stress-related hormone synthesis and signal transduction, and regulating amino acid and sugar metabolism.

**Supplementary Information:**

The online version contains supplementary material available at 10.1186/s12870-022-03785-5.

## Background

With global climate change, the growth and development of plants are often subject to different environmental pressures, such as drought, heat, cold, and high salinity [1]. In particular, as the global temperature rises and the area of dry land continues to increase, drought pressure has gradually become a critical problem facing humankind [2, 3]. Therefore, the identification of a strategy for coping with drought stress is of great significance to the sustainable development of global agriculture.

The root system of peach trees is shallow, and drought stress will adversely affect peach production. Drought stress is one of the major abiotic stresses limiting fruit production and quality during the 4–6 week period before harvesting [4], it affects fruit quality and secondary metabolism, increasing phenolic content [5]. And drought significantly reduced sucrose and starch content in peach leaves, the partitioning of newly-fixed carbon was also affected by stress. These changes appeared to originate from the inhibition of photosynthesis induced by drought stress [6, 7]. Drought also affected the activity of peach tree cells, destroy cell membrane stability and affect hormone levels in plants [8, 9].

Drought stress can cause a series of physiological and biochemical reactions in plants, such as the limitation of photosynthesis, enhanced stomatal closure, changes in the cell composition, stimulation of osmotic fluid, and accumulation of reactive oxygen species [10]. Under drought stress, the water balance in a plant is broken, protoplasts are damaged or even destroyed, the cell membrane loses its selective permeability, and small molecular substances such as intracellular inorganic ions and free amino acids flow out. These effects produce large amounts of free radicals and reactive oxygen species. Because cells cannot eliminate reactive oxygen species within a short period of time, these substances will directly attack and destroy the cell membrane system [11]. Drought stress seriously affects the photosynthesis of plants, and osmotic stress caused by drought can induce the rapid accumulation of abscisic acid in plants [12]. In response to drought stress, plants induce the guard cells to close the stomata, photosynthesis cannot proceed normally, and the growth and development of plants are slowed or even stopped [13]. Plants will exhibit a systematic drought response mechanism through a series of stress responses. Plants can enhance their ability to adapt to drought through various methods, such as water regulation, root regulation, stoma regulation, hormone regulation, osmotic regulation, and redox regulation [14–18].

The content of silicon in the Earth’s crust is close to 28%, which is the second most abundant element in the Earth’s crust [19]. The content of silicon in a plant accounts for 0.1% to 10% of its dry weight [20]. Although silicon is not an essential element, its beneficial effects have been confirmed in rice, sorghum, cucumber and other plants [21–23], particularly under stress conditions [24]. Silicon plays an important role in stimulating allergic reactions in the body; enhances the resistance of plants to diseases and insects; relieves heavy metal stress and ultraviolet radiation; improves salt and drought resistance and tolerance to nutrient deficiency; promotes plant growth; and improves the leaf photosynthesis process and fruit quality.

Silicon alleviates various types of abiotic and biotic stresses, such as drought, cold, salt, heavy metals, plant diseases and insect pests [23, 25]. Under drought conditions, the relative water content, water potential and growth of wheat treated with silicon are higher than those of nontreated wheat, and these increases can change the structure of wheat leaves, reduce transpiration, and thus enhance the drought resistance of wheat [26]. Silicon can be deposited in the plant body and promotes the silicification of leaf epidermal cells, which are firmly bound to the cell wall matrix as a component of the cell wall [27, 28]. Silicon is deposited in the plant body, which reduces transpiration, weakens the bypass pathway of transpiration, and reduces the absorption of sodium ions along with transpiration flow and transport to the ground [29]. Silicon supply can improve the root hydraulic conductivity via amending the root growth and increasing root:shoot ratio along with elevating aquaporin activity and osmotic driving power [30, 31]. Silicon is involved in enhancing stress tolerance by regulating secondary metabolism [32]. It induces the production of antitoxin substances, including phenolic substances such as flavonoids and phenolic acids, in injured plant cells. Silicon induces an increase in the activity of proteases related to the disease process in the tissues of affected plants. In susceptible plants, silicon application can increase the activities of peroxidase, catalase, polyphenol oxidase, phenylalanine ammonia lyase, chitinase, β-glucosidase and other proteases. Positive influence of silicon in enhancing the functioning of antioxidative enzymes as well as the elimination of O^2−^ and H_2_O_2_ could be due to the Si triggered decline in oxidative burst by lessening ion toxicity and accretion of nucleoproteins that provide protection to plants against stress conditions [33]. The response of silicon to adverse stress is related to the production and degradation of polyamines, and polyamines can alleviate the damage of reactive oxygen species under adverse stress through their own synthesis and metabolism [34, 35]. Silicon can regulate the level of stress hormones in plants and significantly downregulate endogenous jasmonic acid, abscisic acid and salicylic acid under salt stress [36]. Silicon provides bacterial wilt disease resistance in tomato, and this resistance is mostly mediated through signalling pathways involving ethylene, jasmonic acid, and/or reactive oxygen species [37].

Peach (*Prunus persica* (L.) Batsch) is a significant fruit crop worldwide and the fourth most extensively produced fruit crop in China. In this study, we found that exogenous silicon can enhance the drought resistance of peach seedlings and thus performed RNA-Seq to analyse the transcription changes of peach seedlings after exogenous silicon treatment under drought stress. Our analysis showed that silicon changed the transcriptome of peach seedlings under drought stress and enhanced the drought resistance of peach seedlings by regulating hormone, amino acid, and sugar metabolism. Silicon may be an elicitor of drought tolerance in peach seedlings. This research might provide a theoretical foundation for the use of silicon in crop production under drought conditions.

## Materials and methods

### Plant materials and treatments

Peach (*Amygdaluspersica (L.) Batsch*) seedlings were used as the test materials, which has high genetic stability, and the tests were performed at the experimental station of Shandong Agricultural University in 2021. Peach seedlings (15 leaves) with consistent growth and that were free of disease were selected for short-term drought stress experiments. Three treatments were set up in the experiment: control (CK; treatment with water), drought treatment (10% PEG; treatment with 10% PEG), and drought treatment plus the application of silicon (10% PEG + Si; treatment with 10% PEG + 0.06 mmol/L Na_2_SiO_3_). A PEG6000 solution was used to simulate drought, sodium molybdate was added to the solution, and the molybdenum concentration was 0.6 mmol/L. For each treatment, 18 seedlings were selected and cultivated in a hydroponic tank equipped with different treatment solutions for 6 h. After the treatment, the morphology of the seedlings was observed, the water use efficiency was determined, the roots and leaves were stained with Evans blue, and samples were collected to determine the antioxidant enzyme activity.

Peach seedlings (5 leaves) with consistent growth and that were free of disease were selected for the continuous drought stress test. Three treatments were set up in the experiment: control (CK; treatment with water), drought treatment (10% PEG), and drought treatment plus the application of silicon (10% PEG + Si). For each treatment, 30 seedlings were selected and planted in pots with quartz sand in a greenhouse with light (20,000 lx) and day/night temperatures of 28 °C/18 °C, and the air humidity is 50%. Drought stress was carried out by controlling the water content of quartz sand in the pots, water content adjustment with 10% PEG solution. The water content of the CK treatment was 60%, and the water content of the PEG% and PEG% + Si treatment groups was 40%. The application of silicon was 0.6 mmol/L Na_2_SiO_3_ solution applied to the roots, a total of 100 mL, water as a control. After 10 days of treatment, the peach seedlings were observed, the photosynthetic rate was determined, the stomatal state was observed, and root growth was analysed. In addition, samples of roots and leaves were collected, quick-frozen in liquid nitrogen, and stored at -80 °C for the determination of hormones, amino acids and carbohydrates.

### Preparation of peach seedling tissues for RNA-Seq

After 10 days of continuous drought stress treatment, samples were collected from the drought-treated and drought + silicon-treated groups for RNA-Seq. Peach seedlings that randomly selected were rinsed with sterile water, and leaves and roots were clipped for analysis. Three peach seedlings from each treatment were blended as a biological replicate, and 3 replicates of each treatment were included in the study. The samples were promptly frozen in liquid nitrogen and maintained at a temperature of -80 °C until analysis.

### Evans blue staining examination

Evans blue staining examination has been confirmed as a reliable method for microscopic cell death determination. We used that method to determine cell death, as previously described by Baker and Mock, with slight changes. Nine root tips and leaves from each treatment were selected for staining. samples were vacuum infiltrated in 0.1% Evans blue (w/v) solution for 5 min before staining at room-temperature for 3 to 5 h. The samples were then rinsed in phosphate-buffered saline (PBS) containing 0.05% (v/v) Tween-20. The image was taken using an electron microscope. A darker root and leaf image indicated a lower cell activity.

### Determination of the water use efficiency and photosynthetic rate

On a sunny day, the water use efficiency and net photosynthesis of fully developed functional leaves were measured using the CIRAS-3 portable photosynthetic measurement system (PP-Systems, UK). These measurements were repeated 6 times, and the results were averaged.

### Quality percentage of plant leaves

The functional leaves with the same leaf position were cut, weighed and placed in the room, and weighed once at a fixed time to calculate the percentage change rate of leaf weight. Three replicates were performed for each treatment, and the results were averaged.

### Root architecture analysis

For analysis of the root architecture, the peach seedlings were completely removed, and the roots were thoroughly rinsed. Root architecture parameters (root length, root surface area, root diameter, root volume, root tip number and bifurcated number) were measured using the Professional WinRHIZO root analysis system (Epson V 700).

### Physiological index determination

The content of superoxide anion was determined by hydroxylamine oxidation method; the enzyme activity of superoxide dismutase was measured by nitroblue tetrazolium photoreduction method; the activity of peroxidase was measured by guaiacol method; the activity of phenylalanine ammonia lyase was measured by L-phenylalanine method [38]; silicon content determination using silicon–molybdenum blue colorimetry.

### Measurements of stomatal size

10 days after processing, three leaves of the same developmental stage were selected to determine the size of the stomata. Take the lower epidermis of leaves to make specimens, then observed under 400 × magnification using a Fluorescence Microscope (AXI0, Carl Zeiss,Germany).

### Determination of the hormone content

Abscisic acid, zeatin, auxin, and gibberellin were extracted from the freeze-dried samples and measured [39]. The extracted samples were analyzed by high-performance liquid chromatography (HPLC).It was carried out on Shimadzu having a fluorescence detector (Shimadzu RF-10AXL, excitation and emission, 305-365 nm, respectively) fitted with C18 reverse-phase HPLC column (HP hypersil ODS; diameter and length,4 × 200 mm; particle size, 5 μm; pore size, 120-Å; Agilent Technologie). The flow rate was 1.0 ml/min. The abscisic acid, zeatin, auxin, and gibberellin analyses were repeated thrice.

### Determination of the amino acid content

For analysis of the amino acid composition, the contents of amino acids were determined using a fully automatic amino acid analyser (Biochrom, UK). According to the literature [40], the sample was modified in conjunction with this experiment as follows: (1) After drying and crushing, the sample to be tested was passed through a 100-mesh sieve, accurately weighed to 0.5 g, and placed in a 10-mL centrifuge tube; 10 mL of loading buffer was added, and the sample was extracted with ultrasound for 30 min. (2) The sample was centrifuged at 10,000 r∙min^−1^ for 2 min, and the supernatant was collected. (3) The sample was passed through a 0.22-μm water filter membrane, and 1 mL of the sample was collected.

### Determination of sugars and acids content

In the analysis of the sugars and acids contents, the levels of glucose, fructose, sucrose, citric acid and malic acid were determined by capillary zone electrophoresis (CZE), as previously described [41]. The extracted samples were analysed by capillary chromatography (Beckman P/ACE MDQ).

### RNA extraction and quantitative PCR

According to the manufacturer’s instructions, an RNAprep Pure Plant Kit (Tiangen, Beijing, China) was used for the isolation of total RNA from 0.5 g of samples. The PrimeScript™ RT Kit (Takara, Japan) was used to generate first-strand cDNA according to the manufacturer’s instructions. With a QuantStudio® 3 real-time PCR instrument (Thermo, USA) and three biological and three technical duplicates, we performed a RT-qPCR analysis using SYBR® Premix Ex Taq™ (Takara, Japan) and PpActin as the reference gene in accordance with the manufacturer’s recommendations for all reagents. The relative expression levels were calculated using the 2^−ΔΔCT^ approach [42].

### Library preparation, Illumina sequencing, read mapping and quantification analyses

TRIzol reagent was used for the extraction of total RNA according to the manufacturer’s instructions. A NanoDrop 2000 spectrophotometer was used to assess the RNA purity and quantity (Thermo Scientific, USA). An Agilent 2100 Bioanalyzer was used to check the RNA integrity (Agilent Technologies, Santa Clara, CA, USA). Libraries were then constructed using the TruSeq Stranded mRNA LT Sample Prep Kit (Illumina, San Diego, CA, USA) according to the manufacturer’s instructions. OE Biotech Co., Ltd. Performed the transcriptome sequencing and analysis (Shanghai, China).

The libraries were sequenced using an Illumina HiSeq X Ten platform, and 150-bp paired-end reads were produced. The clean reads were mapped to the peach genome (GCF_000346465.2, NCBI) using HISAT2 [43]. The FPKM value [44] of each gene was calculated using Cufflinks, and the read count of each gene was obtained by HTSeq-Counts. Differential expression analysis was performed using the DESeq (2012) R package [45]. The criteria for substantially differential expression were the following: *P* value < 0.05 and fold change > 2 or < 0.5. To show the expression pattern of genes in the various groups and samples, a hierarchical cluster analysis of differentially expressed genes (DEGs) was performed. Based on the hypergeometric distribution, GO enrichment and KEGG pathway enrichment analyses of the DEGs were performed using R.

### Statistical analysis

The biological and biochemical data are presented as the means ± standard errors (SEs). The significance of the differences between samples was assessed by Duncan’s multiple range tests using SPSS version 20.0. Separation of signifcant means was accomplished using Duncan Multiple Range Test at 0.05 signifcant levels. One-way analysis of variance (ANOVA) was carried out for statistical analyses of multiple experimental groups.

## Results

### Effects of exogenous silicon on the growth and antioxidant ability of peach seedlings under short-term drought stress

**Fig. 1:**
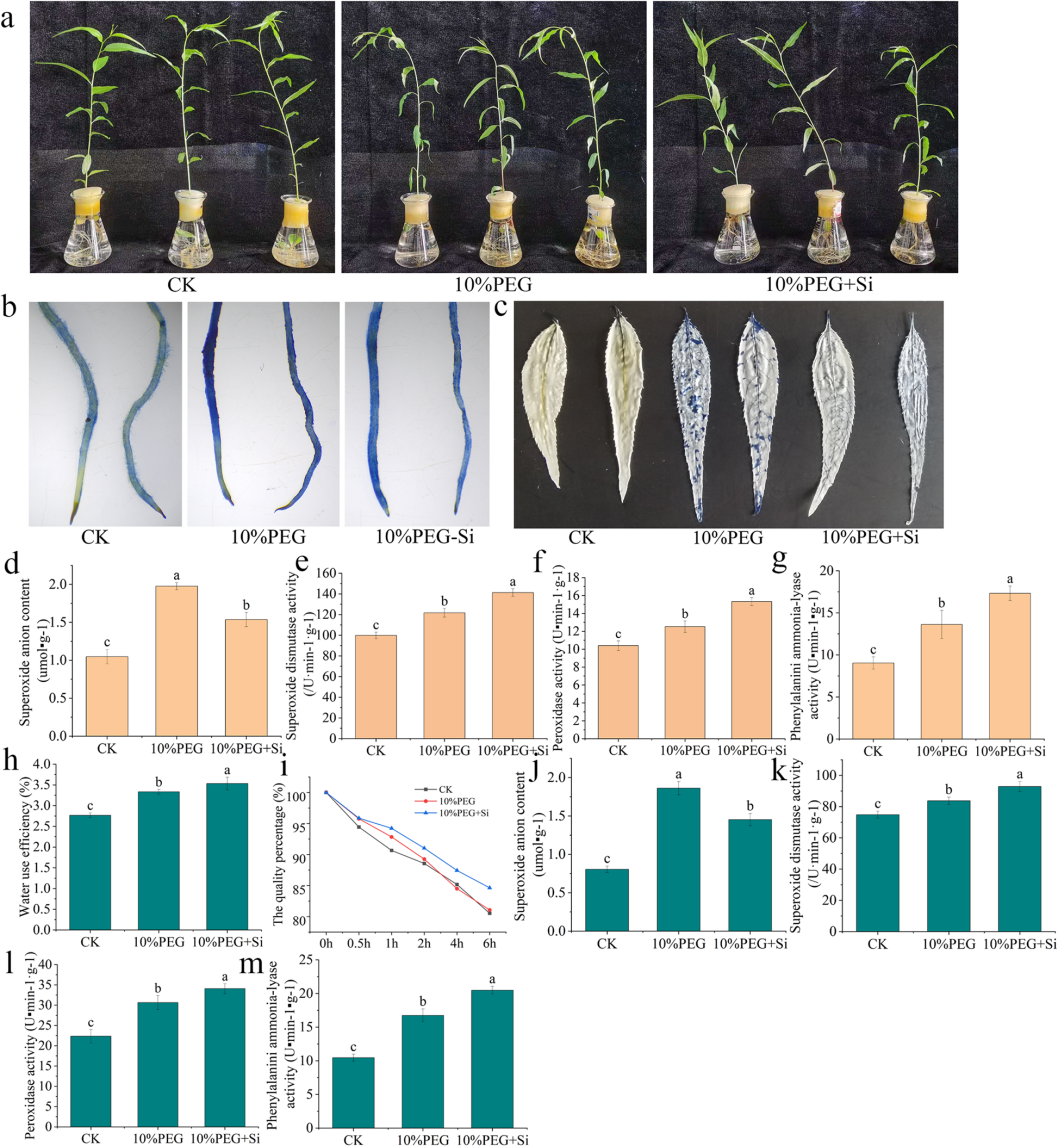
Peach seedling growth status and antioxidant ability under short-term drought stress. **a** Growth status of the peach seedlings. **b** Evans blue dyeing of the roots. **c** Evans blue dyeing of the leaves. The superoxide anion content **d**, superoxide dismutase activity **e**, peroxidase activity **f** and phenylalanine ammonia lyase activity **g** in roots. The water use efficiency **h**, changes in the quality percentage **i**, superoxide anion content **j**, superoxide dismutase activity **k**, peroxidase activity **l** and phenylalanine ammonia lyase activity **m** in leaves.CK: treatment with water; 10% PEG: treatment with 10% PEG; 10% PEG + Si: treatment with 10% PEG + 0.06 mmol/L Na_2_SiO_3_. The bars represent the means ± SEs from three replicates, and each replicate contained six seedlings. Bars with different letters indicate significant differences (*p* < 0.05, Duncan’s multiple range tests)

Under drought stress, the leaf water content of plants decreases with increases in the stress intensity, and the water content can reflect the drought resistance ability of the plants to a certain extent. As shown in Fig. 1a, drought stress induced wilting of the peach seedling leaves and severe water loss compared with the results obtained with the control treatment, and the application of exogenous silicon alleviated this phenomenon.

The leaves and roots of the seedlings were further analysed and stained with Evans Blue. The staining degree of the leaves and roots of the seedlings treated with silicon under drought stress was significantly lighter than that of the seedlings subjected to the drought treatment (Fig. 1b and c), which indicated that the peach seedlings treated with silicon were less damaged. Under drought treatment, treatment with silicon reduced the content of superoxide anion (Fig. 1d), and increased the activities of superoxide dismutase (Fig. 1e), peroxidase (Fig. 1f) and phenylalanine ammonialyase (Fig. 1g) in roots of peach seedlings.The measurement of the water use efficiency of seedling leaves and the quality changes of detached leaves revealed that drought stress can increase the water use efficiency of leaves and slow down the rate of leaf water loss. Silicon strengthened this effect and reduced the leaf water loss (Fig. 1h and 1i). Under drought treatment, treatment with silicon also reduced the content of superoxide anion (Fig. 1j), and increased the activities of superoxide dismutase (Fig. 1k), peroxidase (Fig. 1l) and phenylalanine ammonialyase (Fig. 1m) in leaves of peach seedlings. All these results indicate that silicon reduces the damage induced by drought stress in peach seedlings.

### Effects of exogenous silicon on the growth and hormone content of peach seedlings under continuous drought stress

**Fig. 2:**
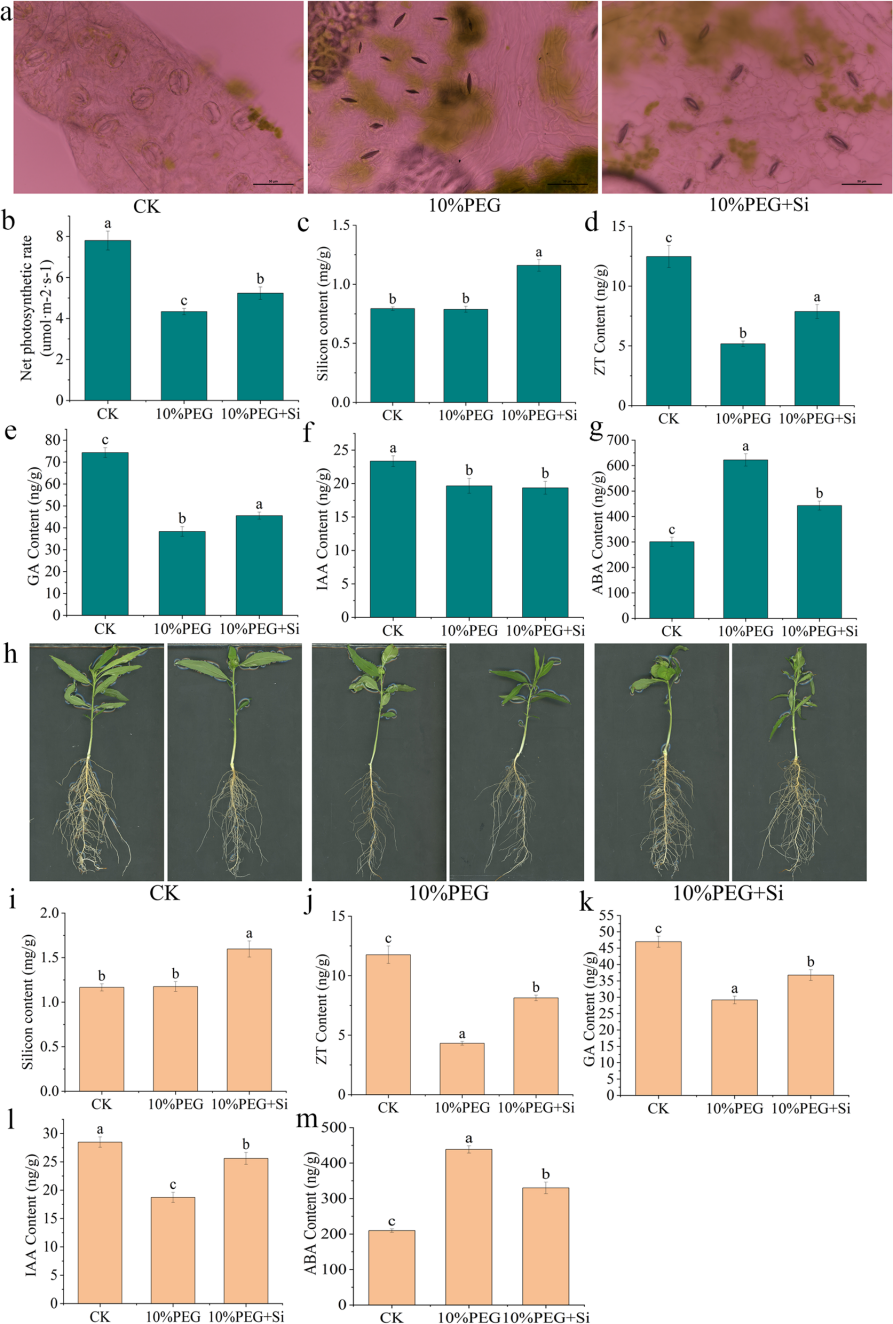
Effects of exogenous silicon on leaf stoma, the root system photosynthetic rate, and the silicon and hormone contents of peach seedlings under continuous drought stress. **a** Opening and closing status of the leaf stomata. The net photosynthetic rate **b** silicon content **c** zeatin content **d** gibberellin content **e** auxin content **f** and abscisic acid content **g** in leaves. **h** Root growth status. The silicon content **i** zeatin content **j** gibberellin content **k** auxin content **l** and abscisic acid content **m** in roots. The values are presented as the mean ± standard error (SE) from three replicates, and each replicate contained six seedlings. Bars with different letters indicate significant differences (*p* < 0.05, Duncan’s multiple range tests)

To further study the role of silicon in drought, we subjected peach seedlings to continuous drought treatments to observe the effects of exogenous silicon treatment on their growth. After 10 days of treatment, we found that exogenous silicon treatment increased the drought resistance of peach seedlings, and further observed the stomata of leaves. Microscopic observations revealed that drought stress caused the leaf stomata to close. Based on the photographs, drought treatment closed the peach leaf stomata (Fig. 2a), decreased the net photosynthetic rate of the leaves (Fig. 2b), and weakened the nutrient production capacity compared with the results obtained with the control treatment. Under drought stress, exogenous silicon increased silicon content in leaves (Fig. 2c), and alleviated the adverse effect of drought stress on peach seedlings, as demonstrated by reductions in both the degree of leaf stomatal closure and the decrease in the net photosynthetic rate. Hormones in leaves were tested and the results showed that drought stress significantly decreased zeatin (Fig. 2d), gibberellin (Fig. 2e) and auxin (Fig. 2f) content and increased abscisic acid content (Fig. 2g) in leaves. However, under drought stress, the silicon treatment reduced the abscisic acid content of leaves by 28.80% (Fig. 2g). Excessive accumulation of abscisic acid induces stomata closure, and the effect of silicon on the stomata may be realized by abscisic acid.

An analysis of the underground parts of peach seedlings revealed thatdrought inhibited the growth of plant roots and reduced root activity (Fig. 2h and Table 1), and exogenous silicon treatment increased the silicon content (Fig. 2i) and alleviated the adverse effect of drought stress in roots. Compared with the results obtained with the control treatment, drought treatment significantly decreased the total root length, total surface area and average diameter of the plant roots (Table 1). The results also showed that silicon promoted the growth of peach seedling roots, particularly the development of lateral roots, under drought stress. The number of lateral roots, root tips and forks of peach seedlings treated with silicon were significantly higher than those obtained with the other treatments (Table 1). The development of lateral roots is closely related to the hormone levels, and our test results verify this fact. Drought stress decreased the contents of ZT, GA, and IAA in the peach roots (Fig. 2j, k and l) but increased the content of ABA (Fig. 2m). However, the silicon treatment increased the contents of ZT, GA and IAA in the seedlings, decreased the content of ABA, and elevated the ZT/ABA, GA/ABA, IAA/ABA ratios compared with the results obtained with the 10% PEG treatment. Changes in hormone levels may be an important factor through which silicon regulates the occurrence of lateral roots under drought stress.

**Table 1:** The root architecture analysis of peach seedlings under continuous drought stress

**Treatment**	Total Length (cm)	Total surface area (cm^2^)	Average diameter (mm)	First-level lateral root	Tips	Forks	Root activity (ug·g^−1^·h^−1^)
CK	147.30 ± 5.02a	12.01 ± 0.41a	0.49 ± 0.03a	80.33 ± 6.03b	103.00 ± 6.08b	63.67 ± 8.96b	76.50 ± 2.52a
10%PEG	101.26 ± 4.94c	9.23 ± 1.46b	0.32 ± 0.02b	67.33 ± 4.04c	89.33 ± 3.79c	59.33 ± 6.11b	52.83 ± 3.37c
10%PEG + Si	131.25 ± 9.56b	11.41 ± 0.68a	0.28 ± 0.02b	105.00 ± 7.00a	128.67 ± 6.81a	92.67 ± 4.73a	60.13 ± 1.99b

Different letters indicate significant differences (*p* < 0.05, Duncan’s multiple range tests)

### Summary of transcriptome sequencing data

We sampled peach seedlings treated with 10% PEG and 10% PEG + 0.06 mmol/L Na_2_SiO_3_ to study the genetic basis for the drought tolerance of peach seedlings exposed to exogenous silicon through RNA-Seq analysis. The RNA-Seq data from three biological duplicates of each sample yielded an average of 44–50 million rawreads. We acquired an average of 43–49 million clean readsafter filtering out low-quality reads; more than 92% of the sequencing quality was Q30, and the average GC content of each sample was approximately 46%. We were able to correctly map 92% of the genes to the peach reference genome, and these included more than 90% unique mapped reads and fewer than 2.7% duplicated mapped reads (Table S1). Overall, the quality of the RNA-Seq data enables further investigation.

### Differentially expressed genes between seedlings treated with silicon and control seedlings under drought stress

**Fig. 3:**
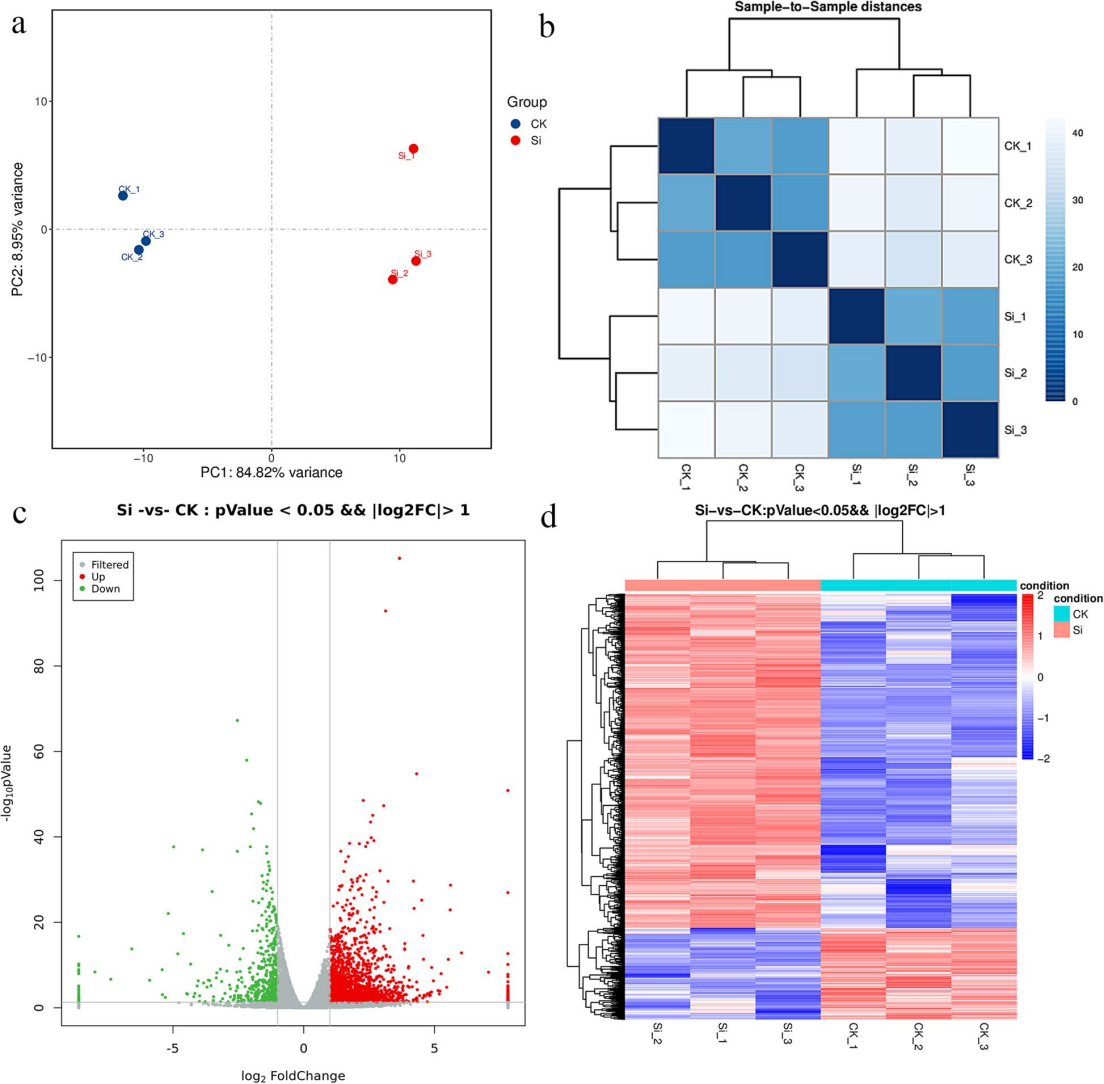
Differentially expressed genes (DEGs) and correlations among various treatment samples. **a** Principal component analysis (PCA) of gene expression levels based on the transcriptomes of three biological replicates treated with exogenous silicon or water under drought stress. **b** Pairwise Pearson’s correlation coefficients (PCCs) of gene expression levels based on the transcriptomes of three biological replicates treated with exogenous silicon or water under drought stress. **c** Numbers of DEGs from the comparison of the exogenous silicon and control treatments under drought stress. **d** Hierarchical cluster map of the DEGs based on the RPKM values. CK: 10% PEG treatment; Si: 10% PEG + 0.6 mmol/L Na_2_SiO_3_ treatment; three biological replicates of each treatment were included in the analysis

To study the difference between the exogenous silicon and control treatments on the gene transcription profiles of peach seedlings under drought stress, we performed a principal component analysis (PCA) of the transcriptome data, and based on the FPKM values, we calculated Pearson correlation coefficients (PCCs) among all expressed genes in all the samples (Fig. 3a and b). Both the PCC and PCA results showed that the samples subjected to the same treatment were close to each other, which indicated that the overall transcription levels of the samples subjected to the same treatment were similar. However, the transcription levels of the samples subjected to different treatments are significantly different, which indicated that under drought stress, the application of exogenous silicon will significantly change the gene transcription profile of peach seedlings. The log2 (fold change) value was used for the hierarchical clustering of the DEGs. In this study, the results demonstrated that the RNA-Seq data exhibited a high level of repeatability across three biological replicates. The differential expression analysis identified a total of 2275 genes that respond to silicon under drought stress, and 1787 and 488 of the genes were up- and downregulated, respectively (Fig. 3c and d).

### GO and KEGG enrichment analyses of differentially expressed genes

**Fig. 4:**
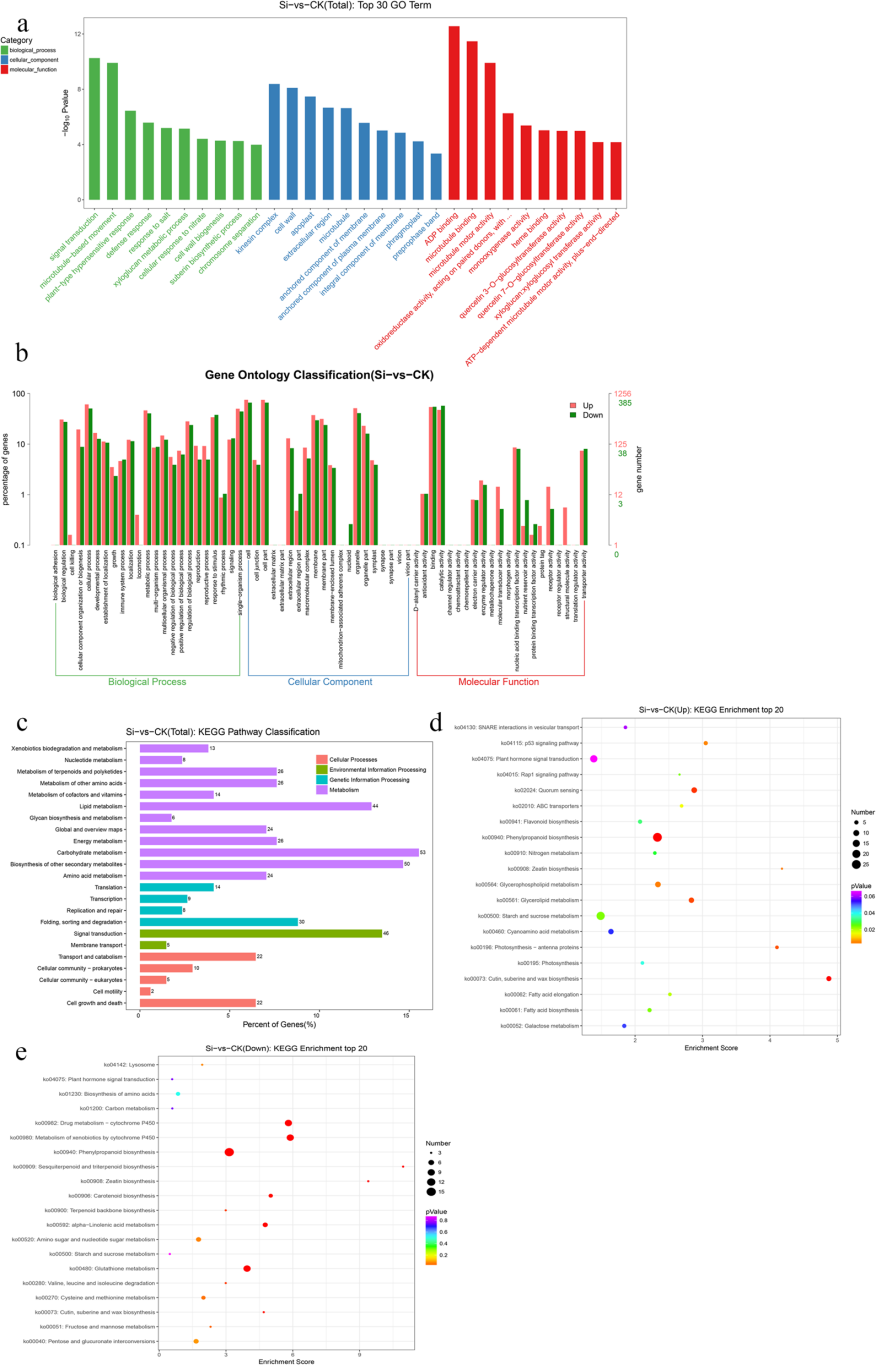
GO and KEGG enrichment analyses of DEGs. **a** Top 30 GO entries that were significantly enriched in DEGs identified from the Si (10% PEG + 0.6 mmol/L Na_2_SiO_3_) vs. CK (10% PEG) comparison. **b** Comparison of the distribution of upregulated and downregulated DEGs at GO level 2. **c** Classification of KEGG pathways enriched in the DEGs identified from the Si (10% PEG + 0.6 mmol/L Na_2_SiO_3_) vs. CK (10% PEG) comparison. **d** Top 20 KEGG entries that were significantly enriched in DEGs (top) identified from the Si (10% PEG + 0.6 mmol/L Na_2_SiO_3_) vs. CK (10% PEG) comparison. **e** Top 20 KEGG entries that were significantly enriched in DEGs (down) identified from the Si (10% PEG + 0.6 mmol/L Na_2_SiO_3_) vs. CK (10% PEG) comparison

To better understand the function of each DEG, a GO enrichment analysis of the DEGs was performed, and their functions were described. The results showed that a total of 2023 DEGs were annotated with 2023 GO terms, and we screened and classified these terms. According to the functional classification, we divided the GO into three levels: level 1, level 2 and level 3. Level 1 contains three items, namely, biological process, cellular component, and molecular function; level 2 contains 64 items, including biological adhesion, cell, and binding; and level 3 contains tens of thousands of items used for conventional enrichment. According to the top 30 GO enrichment terms (Fig. 4a) and the comparison of the distributions of upregulated and downregulated DEGs at GO level 2 (Fig. 4b), the DEGs were found to be involved in signal transduction, the defence response, salt reaction, potassium ion export, L-ascorbate oxidase activity, oxidoreductase activity, immune system process, nucleic acid binding transcription factor activity and other processes.

In addition, we performed a KEGG enrichment analysis of the main pathways involving the DEGs, and the results showed that a total of 147 pathways were regulated by exogenous silicon under drought stress. These pathways mainly involved cell growth and death, cell motility, signal transduction, amino acid metabolism, carbohydrate metabolism, glycan biosynthesis and metabolism and many other processes (Fig. 4c). The top 20 pathways obtained from the KEGG enrichment analysis showed that plant hormone signal transduction (ko04075), starch and sucrose metabolism (ko00500), biosynthesis of amino acids (ko01230), photosynthesis (ko00195), zeatin biosynthesis (ko00908), fatty acid biosynthesis (ko00061), phenylpropanoid biosynthesis (ko00940), carotenoid biosynthesis (ko00906), glutathione metabolism (ko00480) and other important pathways underwent significant changes (Fig. 4d and e). All of these results indicated that silicon is involved in the regulation of drought resistance in peach seedlings.

### Effects of exogenous silicon on pathways of ROS scavenging in peach seedlings under drought stress

Exogenous silicon affected ROS scavenging pathways in peach seedlings, including phenylpropanoid biosynthesis (ko00940), carotenoid biosynthesis (ko00906) and glutathione metabolism (ko00480) (Table S2). Thirty-one DEGs related to the ROS scavenging pathway were screened, including 20 in the propane biosynthesis pathway, 7 in the carotenoid biosynthesis pathway, and 4 in the glutathione metabolism pathway. Comprehensive analysis showed that silicon promoted the biosynthesis of phenylpropanoid, carotenoids and glutathione, especially the expression of peroxidase synthesis genes in the phenylpropanoid biosynthesis pathway was significantly up-regulated, which was consistent with antioxidant enzyme activity. The results indicated that silicon enhanced the antioxidant capacity of peach seedlings.

### Effects of exogenous silicon on hormone, amino acid, starch and sucrose metabolism in peach seedlings under drought stress

The effect of exogenous silicon on the resistance of peach seedlings involves the hormone pathway. Thirty-nine DEGs implicated in hormone production and signal transduction were discovered and investigated. The 21 DEGs involved in hormone synthesis were found to be related to the synthesis and decomposition of zeatin, abscisic acid, gibberellin, auxin and cytokinin (Table 2). A comprehensive analysis revealed that silicon promotes the synthesis and inhibits the decomposition of zeatin, auxin, gibberellin and cytokinin and inhibits their decomposition and also inhibits the synthesis and promotes the decomposition of abscisin. The 18 DEGs were found to be involved in the abscisic acid, auxin, jasmonic acid and cytokinin signal transduction pathways but were mainly enriched in the auxin and cytokinin pathways (Table S3), which affected cell division and the formation of lateral roots. In addition, silicon affects the metabolism of amino acids and promotes the metabolism of amino acids, particularly the phenylalanine pathway (Table S4). The influence of silicon on sugar metabolism is concentrated in the glucose and fructose pathways, which promote the synthesis of glucose and fructose and the accumulation of sucrose (Table S5).

**Table 2:** DEGs involved in hormone biosynthesis

Gene ID	Gene description	log2FoldChange
		Si vs CK
Zeatin biosynthesis		
LOC18770925	Cytokinin dehydrogenase 3	2.05
LOC18771083	Zeatin O-glucosyltransferase	1.70
LOC18784282	Cytokinin hydroxylase	-1.28
LOC18791298	Adenylate isopentenyltransferase 5, chloroplastic	-1.11
LOC18792684	Adenylate isopentenyltransferase	2.34
LOC18793759	Cytokinin dehydrogenase 5	-1.12
Abscisic acid synthesis		
LOC18766758	NDR1/HIN1-like protein 6	-1.13
LOC18780753	9-cis-epoxycarotenoid dioxygenase NCED1	-1.30
LOC18787624	Zeaxanthin epoxidase, chloroplastic	-0.97
LOC18793605	Abscisic acid 8'-hydroxylase 3	1.14
LOC18789983	Probable E3 ubiquitin-protein ligase XERICO	0.71
LOC18768553	Abscisic acid 8'-hydroxylase 4	3.43
Gibberellin biosynthetic		
LOC18781199	Gibberellin 2-beta-dioxygenase 1	-0.63
LOC18786980	Gibberellin 2-beta-dioxygenase 3	2.41
LOC18793977	Gibberellin 2-beta-dioxygenase 6	1.38
LOC18791984	Homeobox protein ATH1	2.56
LOC18786758	Gibberellin 20-oxidase-like protein	1.24
Auxin synthesis		
LOC18782484	Protein REVEILLE 1	2.07
LOC18785562	Anthranilate synthase alpha subunit 1,	3.62
Cytokinin biosynthetic		
LOC18779141	Probable cytokinin riboside 5'-monophosphate phosphoribohydrolase LOGL5	1.47
LOC18770993	Cytokinin riboside 5'-monophosphate phosphoribohydrolase LOG7	1.97

A value lower than 0 indicates downregulation, and a value higher than 0 indicates upregulation. CK: 10% PEG; Si: 10% PEG + 0.6 mmol/L Na_2_SiO_3_

### Effects of exogenous silicon on the amino acid and polysaccharide contents of peach seedlings under drought stress

**Fig. 5:**
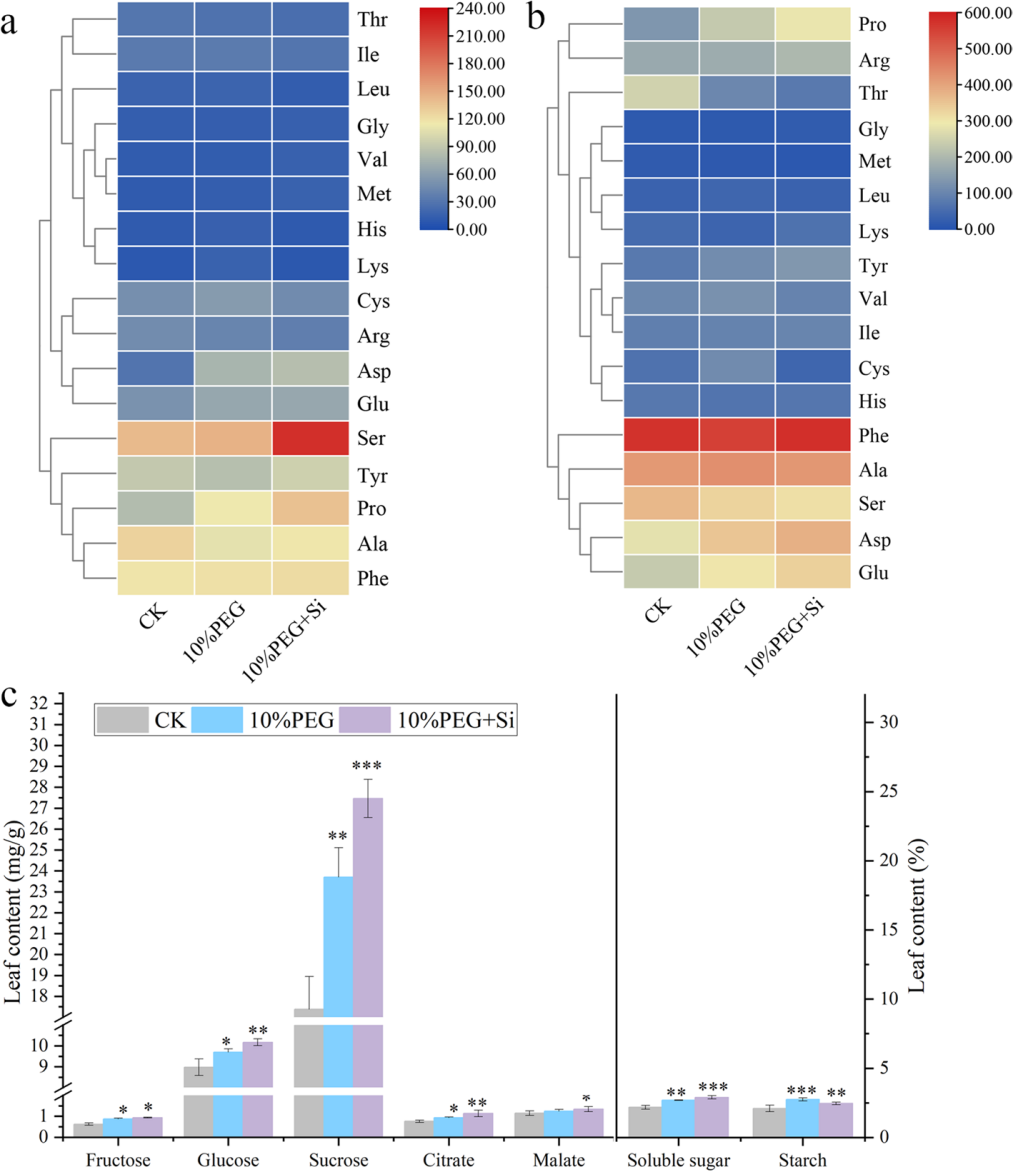
Effects of exogenous silicon on the amino acid and carbohydrate contents of peach seedlings under continuous drought stress. **a** Hierarchical cluster map of the amino acid contents in roots. **b** Hierarchical cluster map of the amino acid contents in leaves. **c** Contents of different types of polysaccharides. The bars represent the means ± SEs from three replicates, and each replicate contained six seedlings

The KEGG enrichment analysis showed that the regulation of silicon on the drought resistance of peach seedlings involves amino acid metabolism, metabolism of other amino acids, glycan biosynthesis and metabolism and carbohydrate metabolism. Thus, we compared the contents of various amino acids and carbohydrates in peach seedlings under drought stress. The results showed that drought stress caused significant changes in the amino acid contents of peach seedlings, increased the total amino acid content, and upregulated the contents of aspartic acid, glutamic acid and proline in leaves and roots. Compared with the results obtained with the drought stress treatment, silicon increased the contents of aspartic acid, proline, glycine, phenylalanine, tyrosine and other amino acids in peach seedlings (Fig. 5a and b). The content of these amino acids is closely related to water changes, which can regulate the cell osmotic pressure and the tolerance of plants to drought stress. The sugar content in the leaves also changed significantly (Fig. 5c). Under drought stress, the application of silicon increased the contents of soluble sugar, sucrose, and glucose by 7.37%, 15.85% and 4.90%, respectively, decreased the content of starch by 10.54%, and did not affect the contents of citric acid and malic acid.

### Validation of gene expression by RT–qPCR

**Fig. 6:**
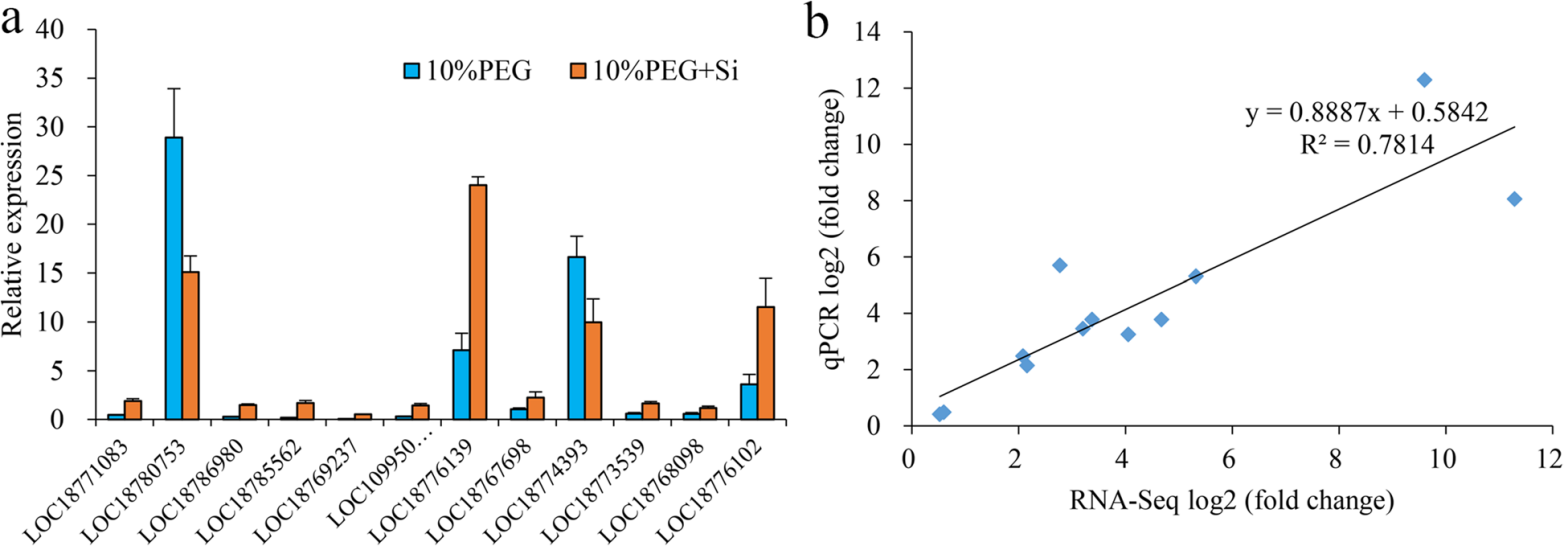
Validation of RNA-Seq data by qPCR. **a** Relative expression of 12 DEGs. **b** Significantly positive correlation of 12 DEGs between RNA-Seq and qPCR data (correlation coefficient of 0.781). The fold change represents the change in expression of each gene under drought stress (Si: 10% PEG + 0.6 mmol/L Na_2_SiO_3_) relative to the respective control (Control: 10% PEG)

To test the reliability of the RNA-Seq data, 12 DEGs were selected for qPCR analysis using TMV resistance protein N (LOC18788576) as a reference gene. The representative genes selected for qPCR analysis were genes involved in hormone biosynthesis, hormone signal transduction, amino acids, starch and sucrose metabolism (Fig. 6a). The RT–qPCR results showed that the expression profiles of most selected genes were similar to those illustrated by the RNA-Seq data (Fig. 6b). This result shows that the RNA-Seq data accurately reflected the transcription level of genes in peach seedlings.

## Discussion

As global climate change intensifies, unfavourable environmental conditions are becoming a major barrier to long-term agricultural sustainability. The utilization of mineral elements as stress mitigators has emerged as the most significant and fascinating part of several innovative tactics meant to protect plants against abiotic stress [46]. As a beneficial element for plant growth and development, silicon can regulate the synthesis of plant endogenous substances and signal transduction to combat adversity caused by abiotic stress and improve plant resistance [47]. Although many studies have shown the beneficial effects of silicon under stress, the exact molecular mechanism of this effect remains unclear. The integration of physiological understanding with omics-scale information highlights that the effects of silicon supplementation on the hormones, amino acids and antioxidant responses of plants under drought stress are key factors that define and improve resilience.

Under drought stress, peach seedlings leaves lose water faster, which causes membrane lipid peroxidation and produces toxic products such as malondialdehyde, hydrogen peroxide and superoxide anion. These substances reduce membrane stability, promote membrane permeability and cause the structure and function of the organelle membrane to be disordered, which causes serious harm to plants [48]. The light saturation point of plants is also decreased, and the percentage of photosynthetic light energy use is reduced [49], the net photosynthetic rate of peach leaves decreased significantly. Drought stress had a serious impact on peach seedlings, as observed by wilted leaves and decreased cell viability. Under drought stress, treatment with silicon reduced the damage caused by drought stress in peach seedlings, decreased the numbers of damaged cells and the superoxide anion content in roots and leaves, and increased the cell viability and antioxidant enzyme activity. In addition, experiments found that silicon improved water use efficiency and reduced leaf water loss. Silicon accumulates in plants and can form covalent bonds with plant cell wall components such as hemicelluloses, pectin and lignin, which may play a crucial role in plant cell wall structure and remodelling. This deposition of silicon results in strong hydrophilicity, can reduce transpiration, decrease the physiological effect of drought on tissue, weaken the transpiration bypass pathway, and reduce the absorption of sodium ions along with transpiration flow and transport to the ground [50, 51]. The improvement in the effects of drought stress obtained with silicon is related to improvement of the internal ion balance and restoration of water resource utilization, this may be the important factor that silicon increases the water content of peach leaves and reduces the rate of water loss in isolated leaves under drought stress. Leaf water loss is reduced and water use efficiency is increased, which can effectively inhibit stomatal closure caused by drought. Silicon can regulate the synthesis of phenols, polyamines, and amino acids and mediate the activity of proteases related to the disease process in response to oxidative stress [23, 52]. Hormones content can also affect the production and/or detoxification process of reactive oxygen species. The RNA-Seq analysis showed that defence reactions, oxidoreductase activity, phenylpropanoid biosynthesis, carotenoid synthesis, glutathione metabolism, ascorbic acid, amino acid synthesis, lipid synthesis and other processes are regulated by silicon. Silicon-mediated biosynthetic pathways of phenylpropanoid, carotenoids and glutathione enhanced the ROS scavenging ability of peach seedlings. Under diverse abiotic stress conditions, a putative function of silicon in improving plant development is related to changes in cellular and biochemical pathways and improved membrane integrity and antioxidant defence systems [46, 53]. These all indicate that silicon can alleviate drought stress damage by reducing oxidative damage and increasing leaf water content.

Plants suffering from drought stress show varying degrees of drought adaptability in terms of the morphological structure and physiological regulation. The root system is an important plant organ that first receives the drought stress signal, and good root system growth conditions are indispensable for enhancing the drought resistance of plants [54, 55]. Experiments have found that silicon promotes the growth of peach seedling roots under drought stress, particularly the development of lateral roots, and increases the length and number of lateral roots. Root development is closely related to hormones. The hormone content in peach seedlings verified that silicon mediates the hormone synthesis pathway. RNA-Seq results have shown that silicon inhibits the synthesis of abscisic acid and promotes its decomposition [36] and enhances the synthesis of auxin, zeatin and gibberellin. Drought stress reduces the content of auxin and cytokinin in plants, silicon alleviates this adverse effect [56], and a higher auxin content can promote the formation of lateral and adventitious roots. Silicon also regulates the signal transduction of auxin and cytokinin and regulates the expression of lateral root development genes. Therefore, silicon promotes the occurrence of lateral roots in a variety of ways, improves the water absorption capacity of roots, and enhances drought resistance. Drought induces the rapid accumulation of abscisic acid in plants. Excessive abscisic acid can easily induce stomatal closure. Therefore, the balance among various hormones plays a more important role in regulating the internal physiological processes of plants [46, 57]. In this study, silicon reduced the rate of stomatal closure caused by drought and reduced the adverse effects of drought on leaf photosynthesis, and these effects were due to the coregulation of multiple hormones. The closure of leaf stomata is jointly regulated by abscisic acid and zeatin, and changes in the IAA/ABA and ZR/ABA ratios also affect the stomatal movement and photosynthetic rate under drought stress. Silicon alleviates stomatal closure under drought stress by reducing ABA accumulation and lowering IAA/ABA and ZR/ABA ratios. The application of silicon enhances tolerance to abiotic stress by changing the homeostasis of peach seedlings hormones and upregulating/downregulating the endogenous hormone levels. A transcriptome analysis found that silicon regulates not only the hormone content of peach seedlings but also the signal transduction pathways related to gibberellin, jasmonic acid and other hormones to improve various defence responses [58–60]. Plant hormone signalling is a cascade reaction that includes signal perception on the cell surface, production of secondary messengers, protein phosphorylation, and ultimately the activation of stress response genes. Silicon regulated the root development and stomatal changes of peach seedlings by regulating stress hormone synthesis and signal transduction.

Water deficits affects the growth of plants, and plants can maintain their water balance and improve their resistance by increasing the absorption of inorganic substances (usually K^+^) and the biosynthesis of compatible solutes [61]. The experimental results showed that silicon increased the amino acid and soluble sugar contents of peach seedlings under drought stress, particularly the contents of proline, phenylalanine, glycine, lysine, glucose and sucrose. A transcriptome analysis also showed that silicon mediates amino acid metabolism, carbohydrate metabolism and glycan biosynthesis and metabolism and thereby improves drought resistance. Compatible solutes such as betaine, sugar and free amino acids protect cells and membrane structures from the harmful effects of a lack of water and help maintain the water balance inside plants [62]. The tolerance of plants to water deficit is positively related to the accumulation of osmotic substances, which are considered adaptive substances used by plants to regulate osmotic regulation and protect various cellular components. The increased content of these substances alleviated the oxidative damage of peach leaves and improved the water retention capacity. Osmotic fluid participates in the drought tolerance of plants by modifying osmotic regulation, removing ROS, maintaining membrane integrity, and altering various proteins and enzymes [63]. Under drought conditions, silicon supplementation reduces the osmotic potential of plant roots without affecting the water content, which indicates that soluble sugars and amino acids (such as alanine and glutamic acid) play a role in osmotic regulation [35, 64]. Exogenous silicon increases the silicon content of peach seedlings by 30%-50%, the osmotic adjustment caused by the application of silicon under drought conditions can also be attributed to the deposition of silicon in the cytoplasm, which results in the formation of high-molecular-weight silicon complexes in the vacuoles of plant cells [65]. In summary, silicon alleviates plant drought stress through osmotic adjustment.

## Conclusion

As an effectual quasi-essential element, silicon plays an important role in improving the drought resistance of peach seedlings. This study showed that exogenous silicon alleviates the damage to peach seedlings induced by drought stress as well as the adverse effects on growth and development, the increases in oxidative damage, the acceleration of water loss, and the decrease in photosynthesis caused by drought. Silicon reduces drought stress damage through various mechanisms, including increasing the accumulation of silicon in peach seedlings, improving antioxidant enzyme activity, improve plant water use efficiency, regulating the synthesis of compatible solutes, mediating hormone synthesis and signal transduction pathways, regulating the ratio and balance of hormones. Our results show that silicon increases the drought resistance of plants through a variety of physiological and biochemical reactions.

## Supplementary Information

**Additional file 1: Table S1.** Mapping statistics of the RNA-Seq data for three replicates in each treatment. **Table S2.** DEGs involved in ROS scavenging. **Table S3.** DEGs involved in hormone signal transduction. **Table S4.** DEGs involved in amino acids metabolism. **Table S5.** DEGs involved in starch and sucrose metabolism.

## Data Availability

The datasets used and/or analyzed during the current study are available from the corresponding author on reasonable request.
