# Supplementary material for: Silicon enhances the drought resistance of peach seedlings by regulating hormone, amino acid, and sugar metabolism
Source: BMC Plant Biol. 2022 Sep 1;22:422. doi: 10.1186/s12870-022-03785-5 (PMC9434905; doi:10.1186/s12870-022-03785-5)
Supplement: Supplementary file 1 — Additional file 1: Table S1. Mapping statistics of the RNA-Seq data for three replicates in each treatment. Table S2. DEGs involved in ROS scavenging. Table S3. DEGs involved in hormone signal transduction. Table S4. DEGs involved in amino acids metabolism. Table S5. DEGs involved in starch and sucrose metabolism. [file 12870_2022_3785_MOESM1_ESM.docx]

**Supplementary Material**

Tabel S1 Mapping statistics of the RNA-Seq data for three replicates in each treatment

| Sample code | Raw reads | Clean reads | Q30 | GC | Mapped reads | Uniquely mapped | Multiple mapped |
| --- | --- | --- | --- | --- | --- | --- | --- |
| CK1 | 48.17M | 47.08M | 91.55% | 45.94% | 43733671(92.88%) | 42489996(90.24%) | 1243675(2.64%) |
| CK2 | 44.10M | 43.48M | 93.52% | 45.72% | 40107221(92.24%) | 39025562(89.76%) | 1081659(2.49%) |
| CK3 | 50.01M | 49.27M | 93.50% | 45.95% | 46034498(93.43%) | 44686672(90.69%) | 1347826(2.74%) |
| Si1 | 45.92M | 44.98M | 91.44% | 45.71% | 41616647(92.51%) | 40500831(90.03%) | 1115816(2.48%) |
| Si2 | 48.91M | 47.87M | 91.68% | 45.58% | 44340041(92.63%) | 43120627(90.08%) | 1219414(2.55%) |
| Si3 | 48.18M | 47.11M | 91.52% | 45.61% | 43483872(92.31%) | 42259362(89.71%) | 1224510(2.60%) |

CK:10%PEG treatment; Si: 10%PEG+0.6mmol/LNa_2_SiO_3_treatment; M: million

Tabel S2 DEGs involved in ROS scavenging.

| Gene ID | Gene description | log2FoldChange |
| --- | --- | --- |
|  |  | Si vs CK |
| Phenylpropanoid biosynthesis | |  |
| LOC18767376 | 4-coumarate--CoA ligase-like 6 | 4.01 |
| LOC18769960 | Peroxidase 53 | 2.57 |
| LOC18770798 | Beta-glucosidase 12 | 2.55 |
| LOC18772399 | Probable mannitol dehydrogenase | 2.61 |
| LOC18773098 | Peroxidase 5 | 1.57 |
| LOC18773423 | Beta-glucosidase 12 | 3.26 |
| LOC18773539 | Beta-glucosidase 12 | 2.51 |
| LOC18773721 | Probable mannitol dehydrogenase | 1.82 |
| LOC18774793 | Probable beta-glucosidase C | 1.47 |
| LOC18775415 | Peroxidase 4 | 1.86 |
| LOC18779847 | Peroxidase 7 | 3.86 |
| LOC18782145 | Peroxidase 4 | 3.06 |
| LOC18784375 | Peroxidase 3 | 1.33 |
| LOC18786011 | Flavonoid 3',5'-methyltransferase | 1.56 |
| LOC18788700 | Peroxidase 11 | 1.74 |
| LOC18789516 | Peroxidase 41 | 2.51 |
| LOC18790823 | Peroxidase 16 | 1.83 |
| LOC18782836 | Cinnamoyl-CoA reductase 1 | -3.34 |
| LOC18774850 | Probable mannitol dehydrogenase | -1.76 |
| LOC18773379 | Probable mannitol dehydrogenase | -2.54 |
| Carotenoid biosynthesis | |  |
| LOC18777459 | Glutathione S-transferase U10 | 3.05 |
| LOC18777994 | Glutamate--cysteine ligase, chloroplastic | 1.70 |
| LOC18783959 | Ribonucleoside-diphosphate reductase small chain | 1.29 |
| LOC18768411 | Probable glutathione S-transferase | 2.13 |
| LOC109949092 | Glutathione S-transferase U10 | -1.26 |
| LOC18766289 | Glutathione S-transferase | -1.42 |
| LOC18766654 | Glutathione S-transferase U7 | -1.14 |
| Glutathione metabolism | |  |
| LOC18768553 | Abscisic acid 8'-hydroxylase 4 | 3.43 |
| LOC18786914 | Carotenoid cleavage dioxygenase 7 | 1.90 |
| LOC18780753 | 9-cis-epoxycarotenoid dioxygenase NCED1 | -1.30 |
| LOC18769054 | Beta-carotene hydroxylase 2 | -8.60 |

A value lower than 0 indicates downregulation, and a value higher than 0 indicates upregulation. CK: 10% PEG; Si: 10% PEG+0.6 mmol/L Na2SiO3.

Tabel S3 DEGs involved in hormone signal transduction.

| Gene ID | Gene description | log2FoldChange |
| --- | --- | --- |
|  |  | Si vs CK |
| Abscisic acid |  |  |
| LOC18783541 | Histidine kinase 2 | 1.71 |
| Auxin |  |  |
| LOC18767398 | Auxin-induced protein 22D | 2.66 |
| LOC18767432 | Auxin-responsive protein SAUR36 | 1.13 |
| LOC18784499 | Auxin-responsive protein SAUR71 | 1.17 |
| LOC18769159 | Auxin-induced protein 15A | 3.01 |
| LOC18769237 | Auxin-responsive protein IAA16 | 1.71 |
| LOC18781394 | Protein TRANSPORT INHIBITOR RESPONSE 1 | 1.68 |
| LOC18792834 | Auxin-responsive protein IAA12 | 1.08 |
| LOC18793200 | Auxin-induced protein 22B | -1.28 |
| LOC18768141 | Auxin-induced protein AUX28 | 3.39 |
| Jasmonic acid |  |  |
| LOC109950039 | Protein TIFY 3B | 1.92 |
| LOC18791864 | Protein TIFY 6B | 1.02 |
| Cytokinin |  |  |
| LOC18771542 | Two-component response regulator ORR10 | 1.16 |
| LOC18768580 | Cyclin-D3-3 | 3.19 |
| LOC18776139 | Two-component response regulator ARR6 | 1.93 |
| LOC18788631 | Two-component response regulator ORR9 | 2.13 |
| LOC18791738 | Histidine kinase 4 | 1.18 |
| LOC18784296 | Histidine-containing phosphotransfer protein 4 | -3.18 |

A value lower than 0 indicates downregulation, and a value higher than 0 indicates upregulation. CK: 10% PEG; Si: 10% PEG+0.6 mmol/L Na2SiO3.

Tabel S4 DEGs involved in amino acids metabolism

| Gene ID | Gene description | log2FoldChange |
| --- | --- | --- |
|  |  | Si vs CK |
| Amino acids |  |  |
| LOC18767698 | Bifunctional aspartokinase/homoserine dehydrogenase 2, chloroplastic | 1.10 |
| LOC109949081 | Betaine aldehyde dehydrogenase 1, chloroplastic | 2.27 |
| LOC18767948 | Branched-chain-amino-acid aminotransferase 2, chloroplastic | -1.04 |
| LOC18768702 | Methylcrotonoyl-CoA carboxylase beta chain, mitochondrial | -1.38 |
| LOC18773898 | Bifunctional 3-dehydroquinate dehydratase/ shikimate dehydrogenase, chloroplastic | 1.38 |
| LOC18774393 | Bifunctional 3-dehydroquinate dehydratase/ shikimate dehydrogenase, chloroplastic | -1.03 |
| LOC18774550 | Phospho-2-dehydro-3-deoxyheptonate aldolase 2, chloroplastic | 1.10 |
| LOC18777994 | Glutamate--cysteine ligase, chloroplastic | 1.70 |
| LOC18779168 | Serine acetyltransferase 3, mitochondrial | -1.30 |
| LOC18782619 | D-glycerate 3-kinase, chloroplastic | 1.01 |
| LOC18785562 | Anthranilate synthase alpha subunit 1 | 3.62 |
| LOC18786755 | 4-coumarate--CoA ligase 2 | 1.03 |
| LOC18787444 | Glyoxylate/hydroxypyruvate reductase HPR3 | 1.32 |
| LOC18788763 | Aminotransferase ALD1 | 1.26 |
| LOC18792295 | Chorismate mutase 3, chloroplastic | 2.00 |

A value lower than 0 indicates downregulation, and a value higher than 0 indicates upregulation. CK: 10% PEG; Si: 10% PEG+0.6 mmol/L Na2SiO3.

Tabel S5 DEGs involved in starch and sucrose metabolism

| Gene ID | Gene description | log2FoldChange |
| --- | --- | --- |
|  |  | Si vs CK |
| Starch |  |  |
| LOC18793797 | Alpha-1,4 glucan phosphorylase L-2 isozyme, chloroplastic/amyloplastic | 1.33 |
| Sucrose |  |  |
| LOC18768098 | Probable sucrose-phosphate synthase 4 | 1.31 |
| LOC18784736 | Acid beta-fructofuranosidase 1, vacuolar | 1.95 |
| Glucose |  |  |
| LOC18770527 | Beta-glucosidase 12 | 1.25 |
| LOC18770798 | Beta-glucosidase 12 | 2.55 |
| LOC18773417 | 4-alpha-glucanotransferase, chloroplastic/amyloplastic | 1.21 |
| LOC18773423 | Beta-glucosidase 12 | 3.26 |
| LOC18773539 | Beta-glucosidase 12 | 2.51 |
| LOC18774279 | Putative pectinesterase/pectinesterase inhibitor 28 | 3.68 |
| LOC18774793 | Probable beta-glucosidase C | 1.47 |
| LOC18776759 | Nudix hydrolase 14, chloroplastic | 1.81 |
| LOC18787238 | Beta-glucosidase C | 1.29 |
| LOC18792862 | Beta-glucosidase 40 | 1.10 |
| LOC18786784 | Beta-glucosidase C | -2.65 |
| Fructose |  |  |
| LOC18776102 | Acid beta-fructofuranosidase 2, vacuolar | 1.79 |
| LOC18781709 | Pectinesterase/pectinesterase inhibitor U1 | 1.10 |
| LOC18788064 | Beta-fructofuranosidase, insoluble isoenzyme CWINV1 | 1.35 |
| LOC18768833 | Probable pectinesterase/pectinesterase inhibitor 41 | -1.26 |
| LOC18789273 | Probable pectinesterase/pectinesterase inhibitor 20 | -4.16 |

A value lower than 0 indicates downregulation, and a value higher than 0 indicates upregulation. CK: 10% PEG; Si: 10% PEG+0.6 mmol/L Na2SiO3.
